# Tumour mutation status and melanoma recurrence following a negative sentinel lymph node biopsy

**DOI:** 10.1038/s41416-018-0088-8

**Published:** 2018-05-14

**Authors:** Nikki R. Adler, Rory Wolfe, Grant A. McArthur, John W. Kelly, Andrew Haydon, Catriona A. McLean, Victoria J. Mar

**Affiliations:** 1 Victorian Melanoma Service, Alfred Hospital, Melbourne, VIC 3004 Australia; 2 School of Public Health and Preventive Medicine, Monash University, Melbourne, VIC 3004 Australia; 3 Divisions of Research and Cancer Medicine, Peter MacCallum Cancer Centre, Melbourne, VIC 3000 Australia; 4 Sir Peter MacCallum Department of Oncology, University of Melbourne, Melbourne, VIC 3000 Australia; 5 Department of Medical Oncology, Alfred Hospital, Melbourne, VIC 3004 Australia; 6 Department of Anatomical Pathology, Alfred Hospital, Melbourne, VIC 3004 Australia; 7 Skin and Cancer Foundation, Carlton, VIC 3053 Australia

**Keywords:** Melanoma, Cancer genetics

## Abstract

**Background:**

A proportion of patients develop recurrence following a tumour-negative sentinel lymph node biopsy (SLNB). This study aimed to explore whether melanoma patients with *BRAF* or *NRAS* mutant tumours have an increased risk of developing disease recurrence following a negative SLNB compared to patients with wild-type tumours.

**Methods:**

Prospective cohort study of melanoma patients at three tertiary referral centres in Melbourne, who underwent SLNB. Clinical, pathological and molecular characteristics and recurrence data were prospectively recorded. Multivariate Cox proportional hazards regression models estimated the adjusted hazard ratio (aHR) and corresponding 95% confidence interval (CI) for the association between mutation status and development of recurrence following a negative-SLNB.

**Results:**

Overall, 344/477 (72.1%) patients had a negative SLNB. Of these, 54 (15.7%) developed subsequent recurrence. The risk of disease recurrence following a negative SLNB was increased for patients with either a *BRAF* or *NRAS* mutant tumour compared to wild-type tumours (aHR 1.92, 95% CI: 1.02–3.60, *p* = 0.04).

**Conclusion:**

Melanoma patients with *BRAF* or *NRAS* mutant tumours had an increased risk compared to patients with *BRAF/NRAS* wild-type tumours of developing disease recurrence following a tumour-negative SLNB. The findings also confirm the importance of continued surveillance to monitor for disease recurrence among SLNB-negative patients.

## Introduction

Sentinel lymph node biopsy (SLNB) is a staging procedure performed for patients with primary cutaneous melanoma to determine the presence of micro-metastatic disease in the sentinel node(s).^1,2^ The status of the sentinel lymph node is a significant prognostic indicator in patients with melanomas greater than 1 mm thick and for patients with melanomas greater than 0.75 mm thick with high risk pathological characteristics.^1^ Indeed, the Multicentre Selective Lymphadenectomy Trial (MSLT-I) determined that sentinel lymph node status is the strongest predictor of disease recurrence in patients with intermediate-thickness and thick primary melanomas.^2^

Nonetheless, a proportion of patients develop locoregional and/or distant recurrence following a tumour-negative SLNB. This may be due to SLNB false negativity or direct haematogenous dissemination without intralymphatic or nodal metastasis in some patients. Previous studies have demonstrated that certain clinicopathological characteristics, including older age, male sex, head and neck location,^3^ Breslow thickness and ulceration^3,4^ are associated with disease recurrence following a tumour-negative SLNB.

It is well-recognised that 40–50 and 15% of cutaneous melanomas harbour activating mutations of *BRAF* and *NRAS*, respectively.^5–9^ Mutations in *BRAF* and *NRAS* oncogenes are associated with distinct phenotypic and histopathological characteristics.^9–14^ Melanomas harbouring these somatic mutations might be associated with differing tumour biology and behaviour. The relationship between tumour mutation status and disease recurrence following a negative SLNB has not yet been specifically investigated.

The primary aim of this study was to explore if patients with *BRAF* or *NRAS* mutant tumours compared to patients with wild-type tumours have an increased risk of developing disease recurrence following a negative SLNB. A secondary aim was to assess the incidence of subsequent disease recurrence and sites of first metastasis among SLNB-negative patients. An improved understanding of the factors associated with disease recurrence among SLNB-negative patients, including mutational characteristics, is important to individualise surveillance strategies.

## Methods

### Study participants and data collection

This was a prospective cohort study of participants in the Melbourne Melanoma Project (MMP). Patients referred to one of three tertiary referral centres in Melbourne, Australia (Victorian Melanoma Service at The Alfred Hospital, Peter MacCallum Cancer Centre and the Olivia Newton-John Cancer Research Institute at the Austin Hospital) with a histologically confirmed primary cutaneous melanoma diagnosed within six months of presentation between 2010 and 2015 were eligible for enrolment. Patients with uveal melanoma, mucosal melanoma, melanoma of unknown primary site and multiple invasive primary melanomas were excluded. Institutional ethics approval was obtained from the contributing sites (project number 07/38). All patients provided written informed consent prior to inclusion in the study.

Clinical, pathological and molecular characteristics and recurrence data were prospectively recorded. Clinical variables recorded by the treating doctor at the patients’ initial presentation included: age, sex, Fitzpatrick skin type and personal history of melanoma. The primary melanomas of 73% of patients enrolled in the MMP cohort were tested for the presence of a *BRAF* and *NRAS* mutation. Patients without *BRAF* and *NRAS* mutation testing were excluded.

The tumour characteristics that were collected included: date of melanoma diagnosis, anatomical location of the primary tumour, Breslow thickness (mm), Clark level, histologic subtype, mitotic rate (n/mm^2^) and ulceration. Tumour histologic subtype was classified as superficial spreading melanoma (SSM), nodular melanoma (NM) and lentigo maligna melanoma (LMM) and ‘other,’ which represented less common subtypes including, acral lentiginous, desmoplastic, naevoid, balloon cell, spindle cell and spitzoid melanoma. The anatomical location of the primary tumour was classified as upper extremity, lower extremity, head and neck region or trunk.

Patients were included in our analysis of the MMP cohort study if they underwent SLNB and had *BRAF* and *NRAS* mutation testing of their tumour. Patients without clinical evidence of metastasis at diagnosis of their primary melanoma and with tumours greater than 1 mm thick or greater than 0.75 mm thick with high risk pathological characteristics (ulceration and/or ≥1 mitosis) were offered SLNB. The decision to undergo SLNB was ultimately made by the patient after receiving adequate information based on available evidence by their treating doctor(s). SLNB was performed at one of the three tertiary referral centres listed above. Preoperative lymphoscintigraphy and intraoperative isosulfan blue dye injection were used to guide the surgeons to the sentinel lymph node in the standard manner. At each of the institutions, sentinel lymph nodes were sent for routine pathological evaluation with haematoxylin and eosin (H&E) staining and immunohistochemistry (S100 protein and Melan-A) with multiple-level sectioning to detect the presence of melanoma cells by an experienced dermatopathologist. There was an independent centralised pathology review system at one of the included institutions (Victorian Melanoma Service, Alfred Hospital), which accounted for approximately 35% of study participants. The status of the sentinel lymph node was classified as tumour-positive or tumour-negative depending on the presence or absence of metastatic melanoma cells in the lymph node.

Patients were followed up after their SLNB as per routine care by one of the tertiary institutions listed above or by community doctors (i.e. general practitioners or specialists), depending on their stage and disease progression. Postal questionnaires seeking information on disease recurrence were sent to community doctors annually. Patients’ disease progression following the negative SLNB was prospectively recorded. The date and site of detected metastasis were recorded for the initial site of metastasis as well as for all subsequent metastases. Recurrence was categorised as satellite/in-transit, regional lymph node or distant metastasis. The date and cause of death was recorded for all participant deaths. Notification of death was from community doctors, hospital medical records, ‘deceased, return to sender’ letters or family correspondence.

### Mutation testing

Mutation testing was performed on all available tumours, regardless of disease stage, at the Department of Anatomical Pathology, Alfred Hospital, Melbourne, Australia or the Department of Diagnostic Molecular Pathology, Peter MacCallum Cancer Centre, Melbourne, Australia. Haematoxylin and eosin-stained sections of formalin-fixed, paraffin-embedded tissue were reviewed by a pathologist, followed by macrodissection to ensure the percentage of tumour cells was enriched to at least 30%. DNA was then extracted from each sample and checked for adequate concentration. Matrix-assisted laser desorption ionisation time-of-flight (MALDI-TOF) mass spectrometry was used for mutational analyses. DNA quality was evaluated via Eppendorf spectrophotometer. The sample was checked for multiple known mutations in *BRAF* (exon 11 and 15), *NRAS* (exon 2, 3 and 4) and *KIT* (exon 11, 13 and 17) using Sequenom (Agena) Mass ARRAY OncoFocus panel (Version 3). The vast majority of samples were tested by the method described above. A minority of samples were tested with next generation sequencing (Illumina MiSeq) or high resolution melting (HRM) analysis following macrodissection of the paraffin-embedded tumour specimens, as previously described.^6,15^ Other mutations were not routinely tested in this cohort.

### Statistical analyses

All statistical analyses were performed using Stata version 14.2 (StataCorp LP, College Station, Texas, USA) statistical software. Baseline descriptive statistics included proportions, mean or median values, as appropriate by data distribution. Mitotic rate (0, 1–5 and >5/mm^2^) was analysed as an ordinal variable. Age (<50 years or ≥50 years) and Breslow thickness (≤2.0 or >2.0 mm) were dichotomised. Mutation status was analysed in two separate ways; (a) dichotomised as mutant (i.e. tumours harbouring either a *BRAF* or *NRAS* mutation) or *BRAF/NRAS* wild-type, and (b) trichotomised as *BRAF* mutant, *NRAS* mutant or *BRAF/NRAS* wild-type.

The outcome of interest, disease recurrence, was defined as the development of distant, regional lymph node or satellite/in-transit metastasis after a negative SLNB. In addition, the development of both subsequent regional lymph node metastasis and distant metastasis following a negative SLNB were analysed separately. Univariate and multivariate Cox proportional hazards regression models were used to estimate the hazard ratio (HR) and corresponding 95% confidence interval (CI) for the association between mutation status and the development of recurrence following a negative SLNB, without and with adjustment for other clinicopathological covariates known to be related to disease recurrence following a negative SLNB. The same process was followed for patients with a positive SLNB. Effect modification was examined by inclusion of an interaction term between SLNB status and mutation status in models fitted to all patients. Statistical significance was defined as a *p* value < 0.05.

## Results

There were 477 patients in the MMP cohort who underwent SLNB and had *BRAF/NRAS* mutation testing of their tumour from 2010–2015. Among these patients, 133 (27.9%) had a positive SLNB and 344 (72.1%) had a negative SLNB. Following SLNB, patients were followed for a median of 4.5 years [interquartile range (IQR) 3.2–6.1 years] to monitor for subsequent disease recurrence. SLNB-negative patients were followed for a median of 4.8 years (IQR 3.5–6.2 years). There were no significant differences in clinicopathological characteristics (i.e. age, sex, Breslow thickness, mitotic rate, ulceration) between patients who had mutation testing of their tumour and those who did not (data not presented).

### Descriptive statistics

The median age of participants at diagnosis was 56.5 years [range 20.6–90.2 years] and 61.6% of participants were male. Among all patients who underwent SLNB, the primary tumour was located on the trunk in 165 (34.9%) patients, upper extremity in 116 (24.5%) patients, lower extremity in 95 (20.1%) patients and head and neck region in 97 (20.5%) patients. The median Breslow thickness was 2.0 mm (IQR 1.3–3.1 mm). The median mitotic rate was 3 mitoses per mm^2^ (IQR 2–7 mitoses per mm^2^) and 34.5% of primary tumours were ulcerated. The majority (53.9%) of primary melanomas were of the superficial spreading subtype, followed by nodular melanoma (30.6%), lentigo maligna melanoma (4.4%), ‘other’ less common subtypes (6.3%) and unknown subtype (4.8%). Table 1 displays the clinicopathological characteristics of patients by sentinel lymph node status.

**Table 1:** Clinicopathological characteristics of patients who had a positive and a negative sentinel lymph node biopsy

Clinicopathological and molecular characteristics	Positive SLNB patients (%)	Negative-SLNB patients (%)	*p* value
Total number	133 (27.9)	344 (72.1)	
Patient sex			
Male	86 (64.7)	208 (60.5)	0.4
Female	47 (35.3)	136 (39.5)	
Patient age			
<50 years	54 (20.9)	113 (32.9)	0.10
≥50 years	78 (59.1)	231 (67.2)	
Breslow thickness			
≤2.0 mm	55 (42.6)	186 (54.6)	0.02
>2.0 mm	74 (57.4)	155 (45.4)	
Mitotic rate (n/mm^**2**^)			
0	11 (8.3)	34 (9.9)	0.04
1–5	67 (50.4)	210 (61.1)	
>5	55 (41.4)	100 (29.1)	
Ulceration			
No	71 (55.0)	233 (69.5)	0.003
Yes	58 (45.0)	102 (30.5)	
Anatomical location			
Head and neck	23 (17.7)	74 (21.6)	0.11
Trunk	53 (40.8)	112 (32.7)	
Upper extremity	24 (18.5)	92 (26.8)	
Lower extremity	30 (23.1)	65 (19.0)	
Histologic subtype			
SSM	73 (58.9)	184 (55.8)	0.3
NM	40 (32.3)	106 (32.1)	
LMM	2 (1.6)	19 (5.8)	
Other^a^	9 (7.3)	21 (6.4)	
Mutation status			
* BRAF/NRAS* WT	30 (22.6)	115 (33.4)	0.02
* BRAF* or *NRAS* mutant	103 (77.4)	229 (66.6)	

*SSM* superficial spreading melanoma, *NM* nodular melanoma, *LMM* lentigo maligna melanoma, *WT* wild type, *HR* hazard ratio, *CI* confidence interval

^a^Other represents acral lentiginous, desmoplastic, naevoid, balloon cell, spindle cell and spitzoid melanoma

### Tumour mutation frequencies

Among patients who underwent SLNB, 222 (47.6%) were *BRAF* mutant, 105 (22.0%) were *NRAS* mutant and 145 (30.4%) were *BRAF/NRAS* wild-type. Among the 344 patients who had a negative SLNB, 146 (42.4%) were *BRAF* mutant, 83 (24.1%) were *NRAS* mutant and 115 (33.4%) were *BRAF/NRAS* wild-type. Among all *BRAF* mutant tumours, the most common genotype was V600E (70.0%), followed by V600K (22.9%) and less common genotypes (7.1%). The vast majority (94.3%) of *NRAS* mutant tumours had an *NRAS* codon 61 mutation. Only 48 tumours had *cKIT* mutational testing and all were *cKIT* wild-type.

### Sites of recurrence following a negative SLNB

**Fig. 1:**
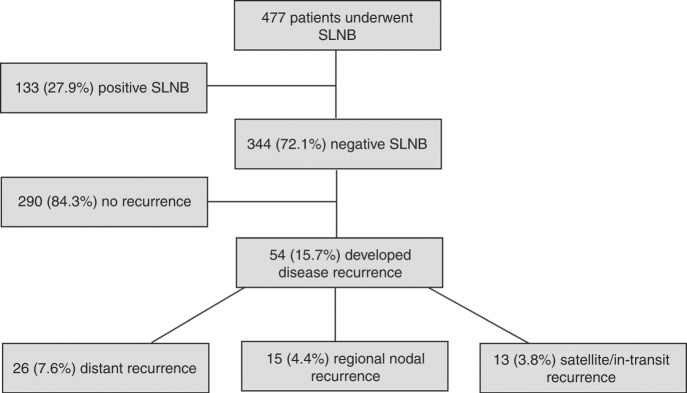
Site of first detected disease recurrence among 344 patients who had a tumour-negative sentinel lymph node biopsy. Figure adapted from Zogakis et al.^26^

Among patients with a tumour-negative SLNB, 54/344 (15.7%) developed subsequent recurrence with a median time to recurrence of 1.8 years (IQR 0.9–3.5 years). Among patients who developed disease recurrence following a negative SLNB, 26 (7.6%), 15 (4.4%) and 13 (3.8%) developed subsequent distant, regional lymph node and satellite/in-transit metastasis as the site of first detected metastasis, respectively (Fig. 1). There were no patients who had multiple sites of recurrence (for example, both satellite/in-transit and distant metastasis) detected concurrently. Of note, 4.4% of patients developed recurrence in the same regional nodal basin as the site of first recurrence despite the fact that it had been found to be tumour-negative at SLNB pathological evaluation.

### Clinicopathological and molecular characteristics associated with disease recurrence following a negative SLNB

Table 2 displays estimated associations of clinicopathological and molecular factors with disease recurrence following a negative SLNB. Older patients (adjusted hazard ratio [aHR] for patients ≥50 years: 2.02, 95% CI 1.00–4.07, *p* = 0.05) and patients with thicker primary tumours (aHR for tumours >2.00 mm thick: 3.08, 95% CI 1.14–3.82, *p* = 0.018) had a statistically significant increased risk of developing disease recurrence among SLNB-negative patients. The associations between both histologic subtype and anatomical location of the primary tumour with disease recurrence did not differ by sentinel lymph node status. When age and Breslow thickness were categorised differently or treated as continuous variables, there is was little difference to the main findings (data not shown).

**Table 2:** Univariate and multivariate Cox proportional hazards regression model estimates of clinicopathological and molecular characteristic associated with the development of disease recurrence in patients following a positive and negative sentinel lymph node biopsy

	Univariate analysis									Multivariate analysis^a^								
Clinicopathological and molecular characteristics	Associations with recurrence following a positive SLNB			Associations with recurrence following a negative SLNB			Comparison of associations in positive vs. negative-SLNB patients			Associations with recurrence following a positive SLNB			Associations with recurrence following a negative-SLNB			Comparison of adjusted associations in positive vs. negative-SLNB patients		
	HR	95% CI	*p* value	HR	95% CI	*p* value	Ratio^b^	95% CI	*p* value	HR	95% CI	*p* value	HR	95% CI	*p* value	Ratio^b^	95% CI	*p* value
Patient sex																		
Male	1.00			1.00			1.00			1.00			1.00			1.00		
Female	0.75	0.41–1.38	0.4	0.78	0.44–1.38	0.4	0.97	0.42–2.24	0.9	0.86	0.46–1.60	0.6	0.82	0.46–1.48	0.5	1.04	0.45–2.44	0.9
Patient age																		
<50 years	1.00			1.00			1.00			1.00			1.00			1.00		
≥50 years	1.33	0.74–2.39	0.3	2.35	1.18–4.69	0.015	0.57	0.23–1.40	0.2	1.06	0.58–1.95	0.9	2.02	1.00–4.07	0.05	0.52	0.21–1.31	0.17
Breslow thickness																		
≤2.0 mm	1.00			1.00			1.00			1.00			1.00			1.00		
>2.0 mm	3.12	1.68–6.54	0.001	2.60	1.46–4.62	0.001	1.27	0.52–3.10	0.6	2.59	1.28–5.27	0.009	2.08	1.14–3.82	0.018	1.24	0.50–3.07	0.6
Mitotic rate (n/mm^**2**^)																		
0	1.00			1.00			1.00			1.00			1.00			1.00		
1–5	1.59	0.37–6.80	0.5	2.50	0.58–10.74	0.2	0.63	0.08–4.97	0.7	0.93	0.21–4.08	0.9	2.03	0.47–8.75	0.3	0.46	0.06–3.60	0.5
>5	2.59	0.61–10.92	0.2	5.71	1.31–24.90	0.02	0.45	0.06–3.55	0.5	1.42	0.32–6.29	0.6	3.62	0.81–16.10	0.09	0.39	0.05–3.09	0.4
Ulceration																		
No	1.00			1.00			1.00			1.00			1.00			1.00		
Yes	1.72	0.96–3.07	0.07	1.65	0.94–2.90	0.08	1.03	0.46–2.33	0.9	0.95	0.52–1.76	0.9	1.15	0.64–2.06	0.6	0.83	0.37–1.87	0.7
Mutation status																		
* BRAF/NRAS* WT	1.00			1.00			1.00			1.00			1.00			1.00		
* BRAF* or *NRAS* mutant	0.74	0.39–1.46	0.4	1.40	0.76–2.59	0.3	0.53	0.21–1.32	0.18	0.71	0.36–1.41	0.3	1.92	1.02–3.60	0.04	0.37	0.15–0.94	0.04

Comparison of the effect of various clinicopathological and molecular characteristics on subsequent disease recurrence in patients with a positive vs. a negative sentinel lymph node biopsy

^a^Multivariate analysis was adjusted for age, sex, Breslow thickness, ulceration, mitotic rate and mutation status.

^b^Ratio of two hazard ratios, HR: the numerator is the HR for stated association in positive SLNB patients and the denominator is the corresponding HR for negative SLNB. *CI* confidence interval, *HR* hazard ratio, *LMM* lentigo maligna melanoma, *NM* nodular melanoma, *SLNB* sentinel lymph node biopsy, *WT* wild type

In the univariate Cox proportional hazards regression model, patients with either a *BRAF* or *NRAS* mutant tumour had an increased risk of developing recurrence following a negative SLNB compared to patients with wild-type tumours (HR = 1.40); however, unadjusted, this did not reach statistical significance (95% CI 0.76–2.59, *p* = 0.3). This association was stronger when adjusted for other known prognostic variables and there was a statistically significant increased risk of disease recurrence following a negative SLNB for patients with either a *BRAF* or *NRAS* mutant tumour compared to those with wild-type tumours (aHR 1.92, 95% CI 1.02–3.60, *p* = 0.04). Therefore, patients with a negative SLNB who harboured a *BRAF/NRAS* mutation experienced a hazard of disease relapse almost twice that of patients with *BRAF/NRAS* wild-type tumours.

When *BRAF* and *NRAS* mutation were analysed separately, patients with *BRAF* mutant tumours compared to patients with *BRAF/NRAS* wild-type tumours had increased risk of developing disease recurrence following a negative SLNB (aHR 2.07, 95% CI 1.05–4.09, *p* = 0.04). There was some evidence to suggest that patients with *NRAS* mutant tumours compared to those with *BRAF/NRAS* wild-type tumours had an increased risk of developing disease recurrence following a negative SLNB (aHR = 1.72, 95% CI 0.80–3.67, *p* = 0.16).

When distant disease recurrence was analysed separately, the results were not conclusive as to whether patients with mutant tumours, compared to patients with wild-type tumours, had an increased risk of developing distant recurrence as the site of first metastasis following a negative SLNB (aHR 1.93 95% CI 0.78–4.82, *p* = 0.16). In addition, our data could not exclude the possibility of a strong positive association between tumour mutation status and the risk of regional nodal recurrence following a negative SLNB (HR 2.04, 95% CI 0.58–7.23, *p* = 0.3) given the size of our cohort.

Interestingly, the association of tumour mutation status with the development of disease recurrence following a SLNB differed depending on the status of the sentinel lymph node (Table 2). That is, the adjusted HR for the association of *BRAF* or *NRAS* mutation with disease recurrence was 63% greater than for patients with a negative than a positive SLNB (*p* = 0.04). That is, the presence of a *BRAF* or *NRAS* mutation had a significantly greater effect at estimating the risk of disease recurrence among patients with a negative compared to positive SLNB. In contrast to this finding for mutation status, the associations held by other covariates (i.e. sex, age, Breslow thickness, ulceration, mitotic rate) with disease recurrence did not show compelling evidence of differing by sentinel lymph node status (Table 2).

## Discussion

The findings of this study suggest that melanoma patients with *BRAF* or *NRAS* mutant tumours have an increased risk compared to patients with *BRAF/NRAS* wild-type tumours of developing disease recurrence following a tumour-negative SLNB. The results of this study contribute to a greater understanding of disease biology among tumours harbouring these somatic mutations and suggest that *BRAF/NRAS* mutant tumours may be more potent drivers of aggressive tumour biology.

The precise role of *BRAF* and *NRAS* mutations in tumour progression has not yet been definitively established. Nonetheless, evidence suggests that *BRAF* and *NRAS* mutations arise early in melanoma pathogenesis and seem to be preserved throughout disease progression.^16^ There is evidence to suggest that *BRAF* mutation is associated with poorer prognostic outcomes in patients with metastatic melanoma.^9,17^ Several studies have also demonstrated that *BRAF* mutation is associated with poorer melanoma-specific survival in patients with early-stage disease.^18,19^ The above results suggest that tumour mutation status might be associated with aggressive tumour biology and might be an important factor involved in disease progression.

Our study also demonstrated that the status of the SLN interacts with the association between tumour mutation status and subsequent disease recurrence. In our study, the presence of a *BRAF* or *NRAS* mutation had a significantly greater effect at estimating the risk of disease recurrence among patients with a negative compared to positive SLNB. Therefore, the effect of tumour mutation status on predicting disease recurrence following SLNB differed by the status of the sentinel lymph node. Of note, the non-statistically significant finding in the univariate analysis and the statistically significant multivariate result may be due to negative confounding, which underestimates the strength of the true association. Furthermore, adjuvant trials have recently shown a survival advantage for patients with stage III disease;^20,21^ therefore, *BRAF* mutation testing to guide clinical decision making will likely occur at an earlier stage and it is important to understand the prognostic implications of this test.

Although SLNB is a powerful predictor of disease recurrence,^2^ it is well-established that a proportion of tumour-negative SLNB patients develop disease recurrence. In our study, 15.7% of tumour-negative SLNB patients developed subsequent disease recurrence. This is consistent with the existing literature, which indicates that 9–24% of SLNB-negative patients develop disease recurrence.^3,4,22–28^

Recurrence following a negative SLNB may be due to multiple factors, one of which is the possibility of primary haematogenous dissemination without lymphogenous spread in a proportion of patients. That is, melanomas may metastasise via the haematogenous route leading to direct distant metastasis in the absence of intralymphatic or nodal disease in a subset of patients.^22,29^ In our study, among patients who developed recurrence following a negative SLNB, 48% developed distant metastasis as the first site of recurrence. This is consistent with the existing literature, which indicates that 31–59% of patients who develop disease recurrence following a negative SLNB present with distant metastasis as the site of first metastasis.^3,4,26,28,30^ Indeed, the MSLT-I trial demonstrated that among all patients who died from melanoma, 48% were ‘node-negative’ (i.e. patients in the observation group without nodal recurrence or in the SLNB true negative group).^2^

This supports the theory that distant metastasis originates from the primary melanoma and that metastatic dissemination is orchestrated in a parallel rather than serial fashion.^29^ The recent results of the MSLT-II study also support this theory as immediate completion lymph node dissection did not improve melanoma-specific survival among patients with sentinel-node metastases compared to observation with frequent nodal ultrasonography and dissection only in patients in whom clinically detected nodal recurrence had developed.^31^ Therefore, the Halstedian hypothesis of contiguous metastasis from the primary tumour through the lymphatics to regional nodes and then to distant sites as the sole mode of disease spread should be rejected.^29^ The model of differential spread proposes that there are multiple independent dissemination pathways with some melanomas able to metastasise only to regional lymph nodes, others able to metastasise only haematogenously and others still able to metastasise haematogenously and via the lymphatic system.^32,33^ Indeed, metastatic patterns vary widely among melanoma patients and the drivers behind the preferential patterns of metastasis among different melanomas remain largely unknown. One explanation is that this may be due to intratumoural heterogeneity and metastatic dissemination that occurs from genetically distinct subpopulations of the primary tumour.^34^

In this study, older patients and patients with thicker primary tumours had an increased risk of developing disease recurrence among SLNB-negative patients. Increasing age, male sex, thicker primary tumours, head and neck location, ulceration and nodular subtype have previously been associated with an increased risk of disease recurrence in SLNB-negative patients.^3,4,28,30^ These factors remain important prognostic markers in patients with a negative SLNB, suggesting that prolonged and more intensive follow-up may be required for this group of high risk patients.

In our study, 4.4% of SLNB-negative patients developed subsequent regional lymph node metastasis. Consistent with our findings, recurrence in the regional nodal basin following a negative SLNB has been reported to occur in 3–11% of patients.^3,4,22,24,35,36^ There are multiple reasons to account for this, including a combination of surgical, pathological and biological factors.^37,38^ Surgical factors related to SLNB false negativity include close proximity of the regional nodal basin to the primary tumour site, multiple lymphatic draining sites and technically challenging surgical sites.^25,39^ Disruption of the regional lymphatics from the previous wide local excision prior to lymphatic mapping may also lead to misidentification of sentinel lymph nodes.^25^ Regarding the pathological factors related to SLNB false negativity, pathological re-evaluation of initially negative sentinel lymph nodes by immunohistochemistry and serial sectioning may detect deposits of occult melanoma cells in a proportion of cases.^22,24,25,37,40^ However, missing occult melanoma in SLNB specimens was unlikely to have played a significant role in our study due to the routine use of immunohistochemistry and serial sectioning and expert dermatopathologist evaluation of all cases.

With respect to biological factors and recurrence in the nodal basin in SLNB-negative patient, it might be the case that in some patients, tumour cells have undergone immune-induced regression in the sentinel node prior to SLNB.^4,25^ In addition, false negativity in the head and neck region may be due to unexpected or aberrant lymphatic drainage or multiplicity in the local lymphatic drainage patterns.^3,41^

The risk of recurrence in the nodal basin previously determined to be SLNB tumour-negative has been shown to be related to increasing Breslow thickness and ulceration of the primary tumour^37^ and advanced age.^36,42,43^ Some propose that age-related traits of the lymphatic system influence metastasising patterns, whereby ageing may cause subclinical stasis contributing to a higher probability of entrapment of melanoma within small lymphatic vessels and/or lymphatic insufficiency.^42^ This might explain the observed lower rates of SLNB positivity among older patients.^44–46^ Some authors suggest that this age-related lymphatic dysfunction may account for a higher rate of SLNB false negativity in older patients.^36,47^

Strengths of our study included the multicentre and prospective nature. Additionally, the MMP database includes comprehensive information on disease recurrence and high-quality longitudinal follow-up data, which compares favourably to other databases. It also contains a rich dataset of phenotypic and tumour-related variables, including mutational data, resulting in a well-characterised cohort of patients with early-stage disease.

Limitations of our study include the median follow-up time of 4.5 years, which would preclude capturing patients with slow tempo disease. Additional studies with comparatively longer observational periods are therefore warranted to definitively capture patients with slow tempo disease. It is also possible that our study was underpowered to detect some covariates as predictors of disease recurrence following a negative SLNB and therefore, larger studies are also warranted. In addition, it is important to consider lead time bias in the detection of disease recurrence as small in-transit and lymph node metastases are more likely to be detected earlier clinically compared to distant visceral metastases of the same size, which may remain asymptomatic. In our multivariate models, we also acknowledge the possibility of residual confounding from unmeasured variables. A further limitation was the availability of only 73% of tumour samples for molecular testing, which might raise the possibility of ascertainment bias. However, there were no significant differences in clinicopathological characteristics between patients who had mutation testing of their tumour and those who did not. The lack of centralised pathology review at all three of the institutions is an additional limitation of our study; nonetheless, routine use of immunohistochemical staining and multiple-level sectioning was performed for all SLNB specimens. Furthermore, we did not test for several important tumour suppressor genes, such as *NF1*, *p53* and *PTEN*.^10^ The relationship between the risk of recurrence following a negative SLNB and concurrent inactivation of important tumour suppressor genes represents an area for future research and may be possible in future cohorts as the cost of sequencing falls.

In conclusion, our study suggests that the development of melanoma recurrence following a negative SLNB might be related to tumour mutation status. If these findings are validated in larger, prospective studies, a role for mutation testing amongst patients with earlier stage disease to guide more intensive surveillance for SLNB-negative patients may be considered, particularly if additional biological parameters combined with *BRAF/NRAS* mutation status allow further enrichment of patients with a high risk of relapse. The findings of our study also confirm the importance of continued surveillance to monitor for melanoma recurrence in SLNB-negative patients, particularly in older patients and patients with thicker primary tumours. In the context of the rapidly evolving landscape of melanoma treatment and the more intensive follow-up of high risk patients, it is important to understand the clinicopathological and mutation predictors of disease recurrence following a negative SLNB.

### Data availability

Data and material supporting the results of this study can be made available by the authors as required.
